# Genetic Analysis of Recently Discovered Least Chub Populations in the Upper Snake River and Bonneville Drainages

**DOI:** 10.1002/ece3.72017

**Published:** 2025-08-22

**Authors:** Eric J. Billman, Michael Terribilini, Cody Smith, Trevor J. Williams, Chance Broderius, Paul Thompson

**Affiliations:** ^1^ Department of Biology Brigham Young University—Idaho Rexburg Idaho USA; ^2^ Independent Researcher Lebanon Ohio USA; ^3^ Utah Division of Wildlife Resources Salt Lake City Utah USA

**Keywords:** Bear River, *Iotichthys phlegethontis*, Lake Bonneville, Snake River

## Abstract

The Least Chub ( *Iotichthys phlegethontis* ) is a small minnow endemic to the Bonneville Basin in Utah. Recently, new populations of Least Chub have been discovered in the upper Snake River in Idaho and in the San Pitch River downstream of Gunnison Reservoir in Utah. The objective of this study was to determine the likely origin of these newly discovered populations of Least Chub. We used genomic data to describe the genetic diversity of Least Chub from extant and refuge populations in Utah and from newly discovered populations. We assessed genetic variation between populations of Least Chub using restriction site‐associated DNA sequencing. Despite low overall genetic diversity, our analyses indicated distinct population genetic structure between drainages and among geographic management units in Utah. Admixture analyses also showed distinct population structuring between groupings with little to no recent admixture. Overall, our results provide evidence that the Idaho Snake River populations and the Utah Gunnison population represent natural, distinct populations of Least Chub. The landscape for Least Chub conservation will need to change as we have provided evidence that the range of Least Chub is double the long‐accepted range for the species. We recommend extensive surveys in both the Bonneville Basin and the upper Snake River drainage to further delineate the distribution of Least Chub and to identify new populations of the species.

## Graphical abstract

Recently, new populations of Least Chub have been discovered in the upper Snake River in Idaho and in the San Pitch River downstream of Gunnison Reservoir in Utah. Overall, our results provide evidence that the Idaho Snake River populations and the Utah Gunnison population represent natural, distinct populations of Least Chub. The landscape for Least Chub conservation will need to change, as we have provided evidence that the range of Least Chub is double the long‐accepted range for the species.

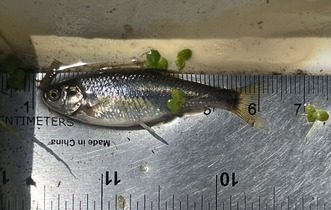

## 1 Introduction

Freshwater fishes exhibit high diversity and endemism because the terrestrial landscape limits dispersal within and among drainage basins (Olden et al. 2010). Historically, range expansions resulted from rare events including stream captures, large‐scale floods, or unintentional movement by terrestrial organisms (Burridge et al. 2006). More recently, species have been introduced beyond their native ranges both intentionally and unintentionally (Leroy et al. 2023). This has resulted in challenges in determining the origin of fish when discovered outside of their native range; that is, were the fish introduced outside of their native range by humans or did the fish expand their range through natural processes ? Population genetic studies have been instrumental in both the study of the biogeography of fishes and the study of biological invasions (Hartman et al. 2023; Kokkonen et al. 2024).

**FIGURE 1:**
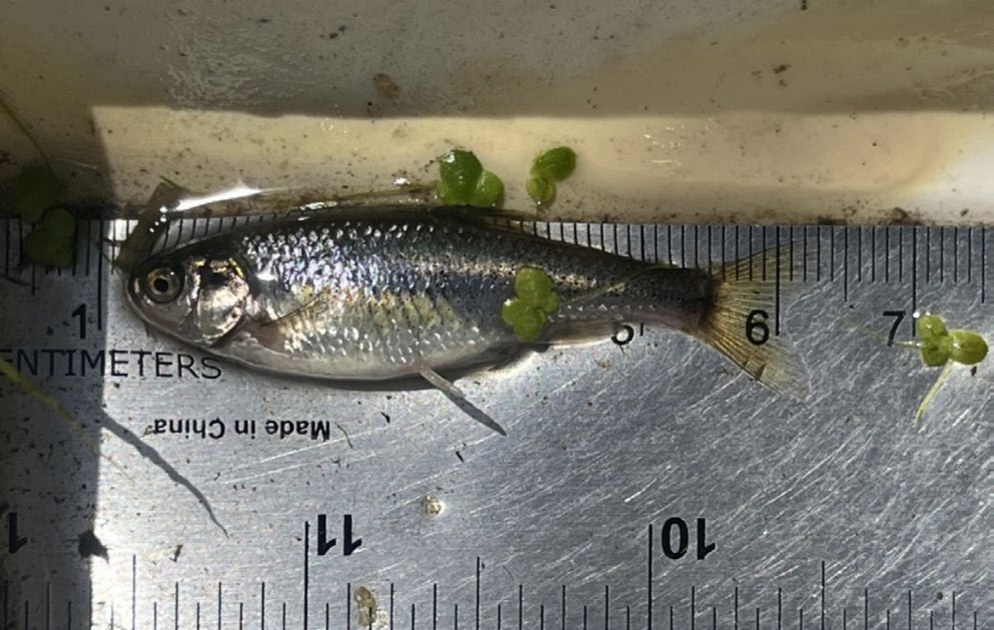
Female Least Chub captured at Harris Ponds, spring ponds tributary to the North Fork Teton River in the Snake River drainage in Idaho.

The Least Chub ( *Iotichthys phlegethontis* ; Figure 1) is a small minnow endemic to the Bonneville Basin in Utah (Hanks and Belk 2004; Bailey et al. 2005). Historically, Least Chub was an abundant species in the Bonneville Basin with a wide distribution that included the Sevier River drainage, the Utah Lake drainage, portions of the Great Salt Lake drainage, and several isolated desert springs (Hanks and Belk 2004). Based on historic fish collections, the habitats of Least Chub included springs, lakes, ponds, marshes, streams, and rivers (Bailey et al. 2005). However, the distribution of Least Chub has been reduced to a few isolated desert spring complexes due to the impacts of invasive species, land use practices, and ground water pumping (Mills et al. 2004; Ayala et al. 2007; Grover 2019). Currently, the Least Chub is managed in three Utah geographic management units (GMU): Wasatch Front GMU, West Desert GMU, and Sevier River GMU (Bailey et al. 2005).

Least Chub have been recently discovered in off‐channel habitats of Henrys Fork Snake River in the upper Snake River drainage of Idaho, but the origin of these fish is unknown (Billman et al. 2023). Least Chub may have experienced a natural range expansion that resulted in fish being transferred between the Bonneville Basin and the Snake River. Multiple connections between the Bonneville Basin and the Snake River over the past several million years have resulted in the transfer of fish species between the Bonneville Basin and the Snake River (Broughton and Smith 2016). Evidence of transfers is apparent in the shared species between the Bonneville Basin and the Snake River (Johnson 2002; Mock et al. 2006; Billman et al. 2010; Unmack et al. 2014; Campbell et al. 2018; Shiozawa et al. 2018). However, prior to their recent discovery, there was no evidence that Least Chub were transferred into the Snake River during these historic connections (Smith et al. 2018). Alternatively, Least Chub may have been introduced into the Snake River more recently through bait‐bucket introductions. The Least Chub is a species of conservation concern; therefore, correctly determining the origin of newly discovered populations in the upper Snake River drainage will be essential for establishing management plans for Least Chub in Idaho. If Least Chub are native to the upper Snake River drainage, the historic range of the species will nearly double in size to include the upper Snake River drainage in Idaho, Wyoming, Utah, and Nevada. Thus, the landscape for conservation of the species has the potential to change drastically depending on the origin of Least Chub in Idaho.

Until recently, researchers were convinced that all known populations of Least Chub in the Bonneville Basin had been identified. However, Least Chub have recently been discovered in the San Pitch River downstream of Gunnison Reservoir (hereafter referred to as the Gunnison population) in the Sevier River GMU (Mecham et al. 2017). This population is the first documentation of Least Chub in a riverine habitat in Utah in over 50 years (Bailey et al. 2005). If the Gunnison population represents a relict population of Least Chub, its discovery provides evidence that additional undiscovered populations may be present in the Bonneville Basin. Genetic analyses will be necessary to determine the relatedness of Least Chub in the Gunnison population with other populations, and to determine if the population has resulted from recent introduction or if the population truly is a natural, distinct population.

The objective of this study was to determine the likely origin of newly discovered populations of Least Chub in the upper Snake River in Idaho and in the San Pitch River in Utah. We used genomic data to describe the genetic diversity of Least Chub from extant and refuge populations in Utah and newly discovered populations. Extant Least Chub populations in Utah have been introduced into discrete, often simple aquatic habitats to establish self‐sustaining populations, termed refuge populations, with the intent of ensuring their genetic persistence in the face of anthropogenic threats (Thompson et al. 2015).

For populations in the upper Snake River, we predicted that Least Chub would show deep divergence from populations in the Bonneville Basin if populations have been isolated since the Pleistocene connections between the Snake River and the Bonneville Basin. Additionally, we predicted that Least Chub from the Gunnison population would closely align with but be distinct from other populations in the Sevier River GMU if this represented a previously undiscovered population. Conversely, we predicted that genetic analyses would demonstrate greater similarity and shared alleles with source populations if Least Chub were introduced into the upper Snake River and the San Pitch River through a bait‐bucket introduction. Results of our study provide context with which the newly discovered populations in Idaho and Utah should be managed.

## 2 Methods

**FIGURE 2:**
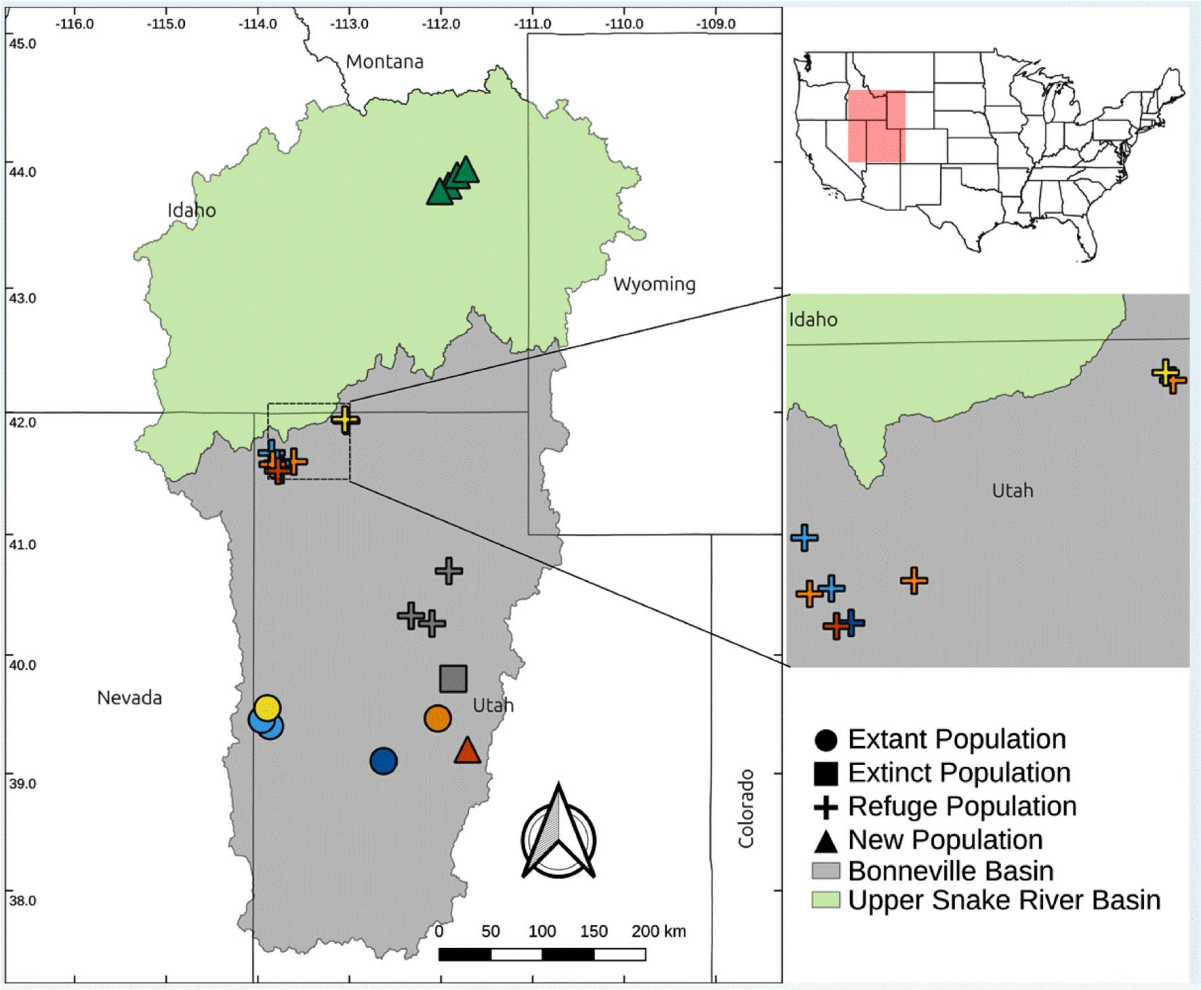
Location of sites where Least Chub were sampled for genetic analyses. In Utah, Least Chub were collected from both extant and refuge populations; in Idaho, Least Chub were collected from four locations spanning the currently documented distribution in the upper Snake River. Populations are colored according to the Admixture analysis with *K* = 7 (see Figure 4).

We acquired samples of Least Chub from extant and refuge populations in Utah and from four sites in the upper Snake River drainage in Idaho (Table 1; Figure 2). In Utah, Least Chub were collected in August–October 2022 using collapsible mesh minnow traps during routine monitoring of populations (LCCT 2010). Mona Springs is a site where Least Chub were extant and from which three refuge populations have been created. However, the population has since been extirpated from Mona Springs. We were able to obtain specimens from Mona Springs that were collected in 2001 for previous genetic analyses of Least Chub (Mock and Miller 2005). For the Gunnison population, we obtained samples from its refuge population (Mud Basin) because we were unable to collect Least Chub from the newly discovered population. We also collected Least Chub using baited, galvanized metal minnow traps in 2022 and 2023 from four locations in the upper Snake River drainage in Idaho (Table 1; Figure 2) including the Cartier Slough Wildlife Management Area where Least Chub were first detected in Idaho (Billman et al. 2023). Redside Shiner (*n* = 3; *Richardsonius balteatus* ) were also collected from Cartier Slough Wildlife Management Area to be used as the outgroup for analyses. For each population or sampling location, we collected a minimum of 8 Least Chub; whole fish or pelvic fin clips were preserved in 95% ethanol.

**TABLE 1:** Number of Least Chub collected from populations in Utah and Idaho.

GMU	Population	Type	Source	Latitude	Longitude	Total samples	Retained samples
West Desert	Bishop	Extant		39.4019	−113.8670	15	14
	Red Knolls	Refuge	Bishop	41.6694	−113.8500	15	12
	Gandy	Extant		39.4549	−113.9562	15	15
	Keg Springs	Refuge	Gandy	41.5853	−113.7903	8	8
	Leland Harris	Extant		39.5532	−113.8962	15	15
	Lower Rocky/Pilot	Refuge	Leland	41.9425	−113.0495	14	14
Sevier River	Clear Lake	Extant		39.1060	−112.6267	15	15
	Willow Pond	Refuge	Clear	41.5281	−113.7468	15	13
	Mills Valley	Extant		39.4664	−112.0349	8	8
	Rosebud Top Pond	Refuge	Mills	41.5984	−113.6070	14	14
	Cluster	Refuge	Mills	41.5763	−113.8386	15	15
	CoPilot	Refuge	Mills	41.9366	−113.0385	15	14
	Gunnison	Unknown		41.5228	−113.7790	15	15
Wasatch Front	Mona	Extinct		39.8001	−111.8662	15	4
	Jail Pond	Refuge	Mona	40.6982	−111.9145	14	14
	Tooele Army Depot	Refuge	Mona	40.3273	−112.3260	14	10
	Big Springs	Refuge	Mona	40.2615	−112.0985	15	15
Snake River	Cartier Slough	Unknown		43.8144	−111.9193	15	15
Snake River	Harris Ponds	Unknown		43.8964	−111.8254	10	10
Snake River	Henrys Fork	Unknown		43.9444	−111.7287	10	10
Snake River	Deer Parks	Unknown		43.7709	−112.0130	10	10
Snake River	Redside Shiner	Outgroup		43.8144	−111.9193	3	3

*Note:* The geographic management unit (GMU) is indicated for extant populations in Utah. Type of population (i.e., extant or refuge) is included for Utah populations; we list the type of population for the Idaho populations and the Gunnison population as unknown because we do not know the origin of these populations. The source population is included for refuge populations. Coordinates for each site are provided in decimal degrees. Total samples indicates the number of samples processed using restriction site‐associated DNA sequencing, while retained samples indicates the number of samples retained for genetic analyses.

We assessed genetic variation between populations of Least Chub using restriction site‐associated DNA sequencing (RADseq; Davey and Blaxter 2010). For whole‐fish specimens, we removed the posterior portion of each fish at the anterior margin of the caudal peduncle; otherwise, pelvic fin clips were used for genetic analyses. Tissue samples (depending on availability, *n* = 8 to 15 per population) were sent to Floragenex Inc. (Portland, Oregon) for processing according to company protocols. In brief, gDNA extraction was completed using ThermoFisher MagMax DNA Ultra 2.0 kits, using Cell and Tissue Extraction Buffer. Genomic DNA quantification was achieved using ThermoQuant‐It assay kits and normalization using Beckman Echo acoustic liquid handling system. Library preparation was completed using SbfI enzyme digest followed by ligation of multiplex RAD adaptors. Spot QA/QC was done prior to paired‐end sequencing (Illumina NovaSeq6000).

### 2.1 RADseq Data Analysis

STACKS v2.64 (Catchen et al. 2011, 2013) was used to identify variable loci among individuals. We followed published guidelines for optimizing the STACKS protocol according to Rivera‐Colón and Catchen (2022). Briefly, we randomly selected two individuals from each population and varied the values of the parameters *M* and *n* (*M* and *n* values were kept identical) to optimize the number of R80 loci (Paris et al. 2017). *M* is the maximum number of mismatches allowed between stacks to form a putative locus, and n is the number of mismatches allowed between individual loci across samples to build a catalog of loci across individuals. We found that the optimal values for M and n were 4. We then included all individual samples with at least 1 million reads and ran the STACKS de novo pipeline. In the final run of the STACKS pipeline, the gt‐alpha parameter was set to 0.01 to include only genotype calls with a *p*‐value of < 0.01. To ensure that our results were consistent across genotyping pipelines, we also genotyped individuals using ipyrad v0.9.96 (Eaton and Overcast 2020) and compared results. As results were largely consistent across pipelines, results shown are those using the STACKS pipeline (ipyrad results available at https://doi.org/10.5281/zenodo.16229886).

### 2.2 Analysis of Population Structure

Using the populations program in STACKS, we filtered the dataset by retaining loci that aligned in > 80% of individuals (command ‐r 0.8) in every population. We also limited the output to only include loci found in at least 6 populations, with a minor allele frequency of at least 0.05. While some populations had a small number of individuals retained (Table 1), we treated refuge and source populations as single population groupings and combined all Snake River populations as a single population group when running the populations program and for downstream analyses. This increased the sample size per population grouping, reducing negative implications of small sample sizes on inferences from the analyses. The refuge populations were all recently established (< 20 years), and preliminary data analysis showed low levels of divergence between source populations and their refuges (mean *F*_st_ < 0.04), warranting this approach. We used the write‐single‐snp option to avoid including linked loci in the downstream analysis. As a final filter, we removed any individuals that did not have a mean depth of coverage of at least 5 from the populations output. These parameters were used in the STACKS population program to calculate population genetics statistics and produce phylip format output files for fixed and variable sites and a vcf file for further downstream analysis.

To investigate genetic variability within and between populations, we conducted Analysis of Molecular Variance (AMOVA; Excoffier et al. 1992). We assessed variability at three levels of organization: (1) between population groups, where population groups consist of a native source population and all the refuge populations derived from it or all the populations sampled within the Snake River, (2) between populations within population groups, and (3) between individuals. We also ran AMOVA between the Gunnison and Mills Valleys populations and between the Mona‐derived and Snake River‐derived populations to specifically test if the newly discovered populations differed significantly from the populations they were most closely related to. We ran AMOVA in R v4.4.2 using the poppr (Kamvar et al. 2015) and pegas (Paradis 2010) packages with a missing threshold set to 0.3 and the within argument set to false. Significance for AMOVA was determined using 1000 permutations.

We assessed population structure between extant and newly discovered populations using both principal component analysis and admixture proportion inference. We conducted principal component analysis (PCA) using the smartpca function in EIGENSOFT v8.0.0 using default parameter values for outlier removal (Patterson et al. 2006; Price et al. 2006). We ran admixture proportion inference using ADMIXTURE v1.3.0 (Alexander et al. 2009; Zhou et al. 2011). To assess the optimal value of *K*, we ran ADMIXTURE's cross‐validation procedure with *K* ranging from 2 to 10 with 20 replicates for each *K* value. After cross‐validation, we ran ADMIXTURE with *K* ranging from 2 to 10, each with 20 replicates to assess variability in latent cluster membership. We assessed variability between model runs and plotted results using Pong v1.5 (Behr et al. 2016).

### 2.3 Phylogenetic Trees

The phylip output files were used to build phylogenetic trees using iqtree v2.0.7 (Minh et al. 2020). The ModelFinder function was used in iqtree to select the evolutionary model. We used the ‐B parameter to run ultrafast bootstrapping with 1000 replicates, and we used Redside Shiner as the outgroup.

## 3 Results

### 3.1 Loci and Polymorphism Statistics

Approximately 2.5 billion reads were produced from the Illumina sequencing runs with an average of about 9 million reads per sample. Following the STACKS protocol and filtering criteria given above, 13,929 variant sites remained. A single SNP per locus was used for all analyses described below.

Genetic statistics based on the SNP results (Table 2) showed that the number of variant sites ranged from 11,335 to 13,696. Nucleotide diversity values, Pi, ranged from 0.04566 to 0.06694, indicating a low level of genetic diversity in Least Chub populations. *F*_is_ values were close to zero for all populations, ranging from −0.00378 to 0.00697, suggesting that the populations are not experiencing inbreeding depression.

**TABLE 2:** Summary of genetic statistics. For each sampling location, we list the number of variant sites, polymorphic sites, private alleles, *p* (frequency of the most frequent allele), Obs Het (observed heterozygosity which is the proportion of individuals that are heterozygotes in the population), Exp Het (expected heterozygosity under Hardy–Weinberg equilibrium), Pi (estimate of nucleotide diversity), and *F*_IS_ (inbreeding coefficient).

Population	Variant sites	Polymorphic sites	Private alleles	*p*	Obs Het	Exp Het	Pi	*F*_IS_
Bishop	11,742	4402	477	0.9586	0.0650	0.0627	0.06411	−0.00198
Clear lake	11,335	4597	447	0.9607	0.0633	0.0608	0.06213	−0.00228
Gandy	12,281	4177	421	0.9555	0.0660	0.0653	0.06694	0.00188
Gunnison	13,676	2382	373	0.9693	0.0472	0.044	0.04566	−0.00345
Leland Harris	13,279	3382	387	0.9653	0.0516	0.0495	0.0505	−0.00265
Mills Valley	13,637	5170	1241	0.9624	0.0581	0.0557	0.05633	−0.00378
Mona	13,522	5135	1212	0.9573	0.0643	0.0624	0.06323	−0.00304
Snake	13,696	4278	1166	0.9589	0.0575	0.0584	0.05913	0.00697

### 3.2 Population Structure

**FIGURE 3:**
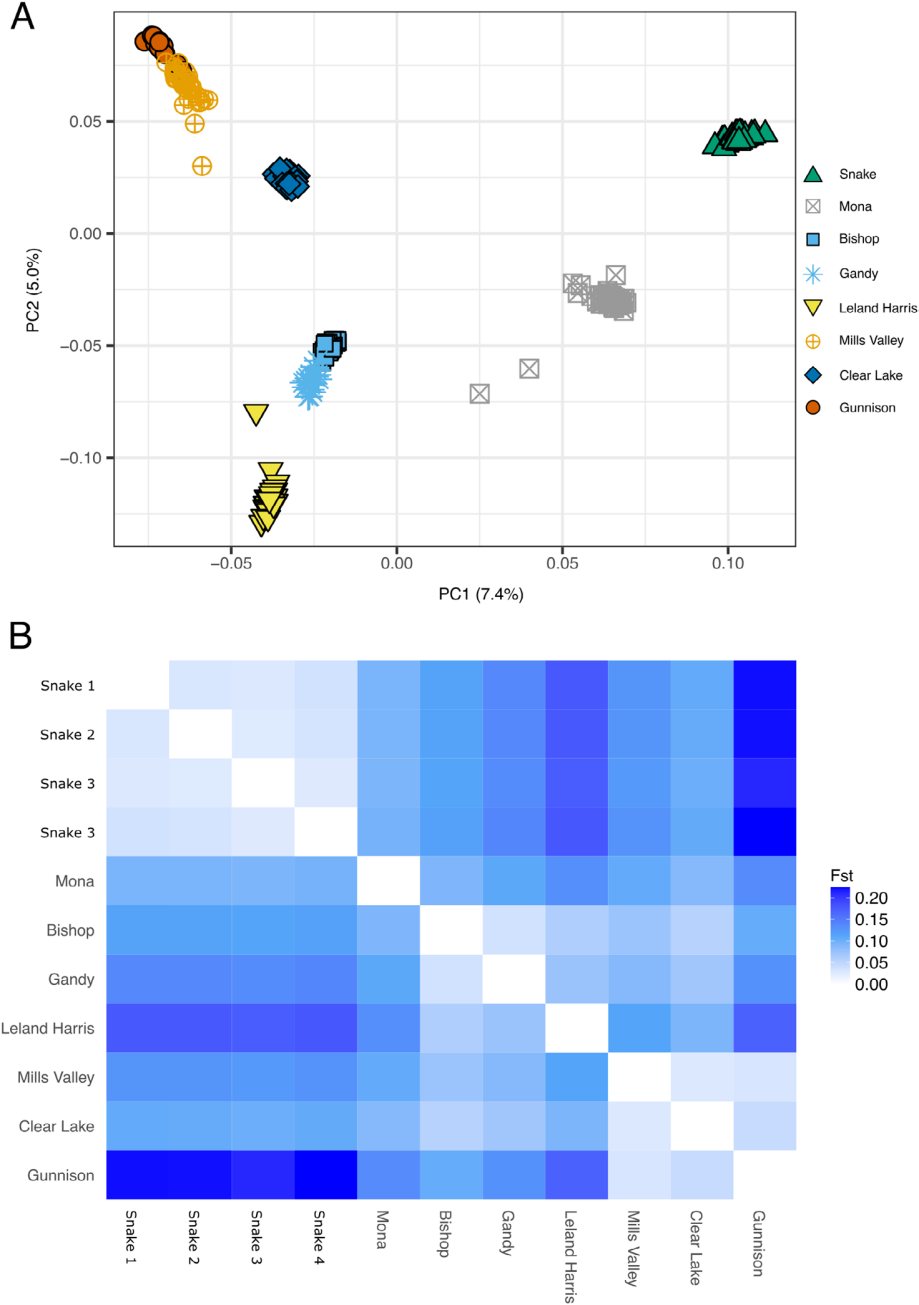
PCA plot and Fst values between populations. (A) PCA plot of genotypic data from each of the sampled populations. After removal of outliers the PCA was constructed from 13,814 SNPs across 225 individuals. Populations are colored according to the Admixture analysis with *K* = 7 (see Figure 4). (B) Heat map showing Fst values between populations. Refuge populations were combined with their source population for analyses and visualization.

Despite low overall genetic diversity, our analyses indicated distinct genetic structure between population groups. Using AMOVA, it was apparent that most genetic diversity (54.9%) was observed between population groups, followed by diversity between individuals within populations (43.3%) and diversity between populations within population groups (1.8%; Table 3). AMOVA between the newly discovered populations and their most closely related population showed significant variation between populations. Higher levels of diversity (54.7%) were seen between populations (rather than within populations) for comparisons between Mona and the Snake River populations. By contrast, there was less variance between populations than within populations for the Gunnison and Mills Valley comparison, though the between‐population variance was still significant. AMOVA results were consistent with PCA clustering of individual populations (Figure 3A). Principal component one separates the Snake River populations from the populations in the West Desert and Sevier River GMUs, while principal component two separates the Sevier River GMU from the West Desert GMU (Figure 3A). Most populations occupied unique positions in PCA space when compared with other populations. The only exceptions were between the Gunnison and Mills Valley populations and the Bishop Spring and Gandy Spring populations (Figure 3). Overall, Fst values showed similar patterns, with the greatest divergences seen between the Snake River populations and the Utah populations (Figure 3B).

**TABLE 3:** Results of AMOVA analyses for all populations as well as between the Mona population and the Snake River populations and between the Gunnison population and Mills Valley population.

Comparison	Percent variation	SSD	MSD	df	*p*
All populations					
Between population groups	54.93%	101719.44	14531.35	7	< 0.0001
Between populations within population groups	1.78%	6667.14	512.86	13	< 0.0001
Between individuals within populations (Error)	43.29%	82628.32	345.73	239	N/A
Mona/Snake River					
Between populations	54.71%	20923.80	20923.80	1	< 0.0001
Within populations (Error)	45.29%	33252.31	386.65	86	N/A
Mills Valley/Gunnison					
Between populations	28.35%	1353.11	1353.11	1	< 0.0001
Within populations	71.65%	5539.44	263.78	21	N/A

Abbreviations: df, degrees of freedom; MSD, mean square deviations; *p*, *p*‐value; SSD, sum of square deviations.

**FIGURE 4:**
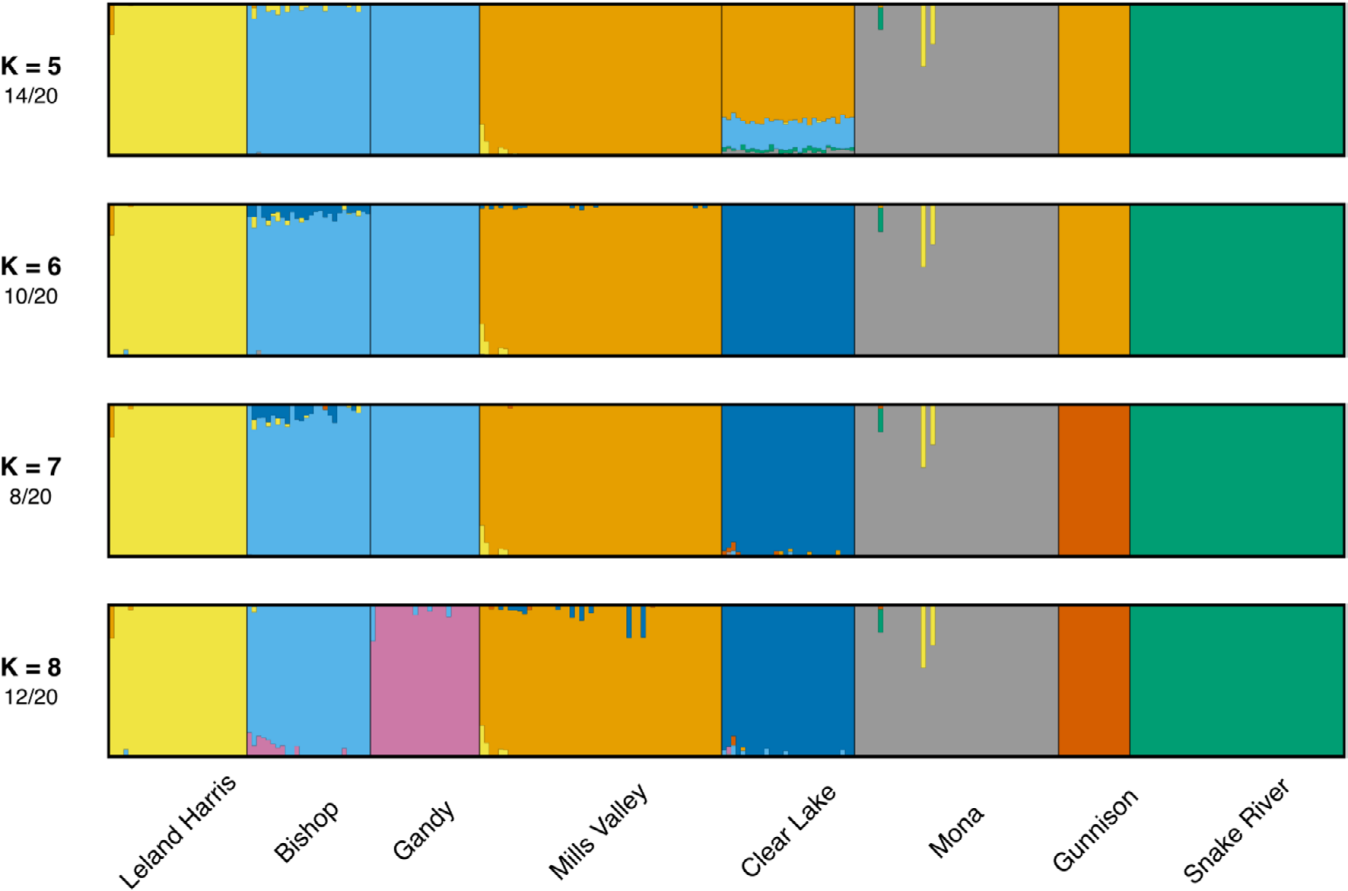
Admixture plot showing population structure for each of the sampled populations. The major modes are shown at K values between 5 and 8, with *K* = 7 having the lowest cross‐validation error. Overall, results are congruent between values of *K* and show little to no recent admixture between populations.

**FIGURE A1:**
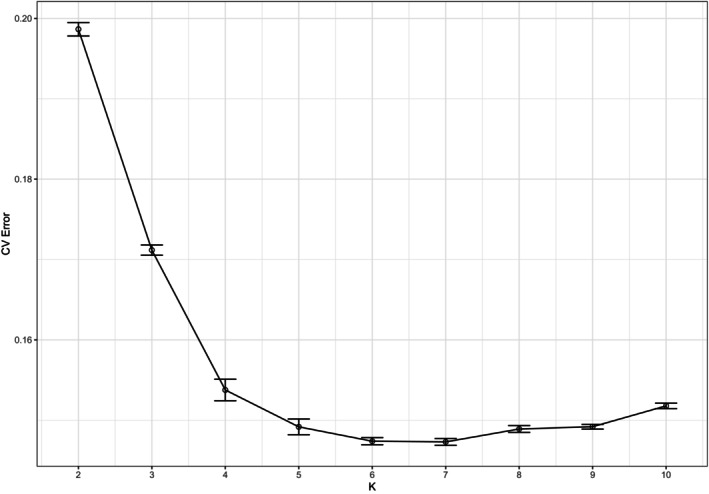
Cross‐validation error for Admixture analyses using output from the Stacks pipeline. *K* = 7 had the lowest cross‐validation error with a mean error of 0.147 and a standard deviation of 0.000428. Cross‐validation was run using 20 replicates for each *K* value.

**FIGURE A2:**
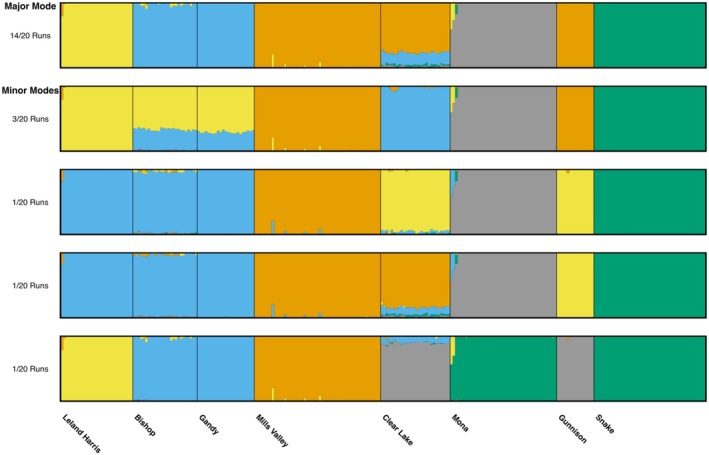
Admixture modes for *K* = 5 using output from the Stacks pipeline. Admixture proportion inference was run using ADMIXTURE v1.3.0 with 20 replicate runs. Variance in latent cluster membership was visualized using Pong v1.5. Modes indicate differences in cluster membership resulting from different runs of the admixture model.

**FIGURE A3:**
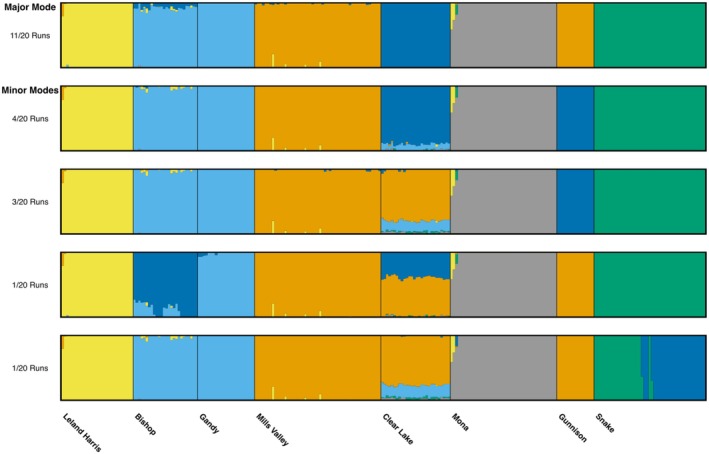
Admixture modes for *K* = 6 using output from the Stacks pipeline. Admixture proportion inference was run using ADMIXTURE v1.3.0 with 20 replicate runs. Variance in latent cluster membership was visualized using Pong v1.5. Modes indicate differences in cluster membership resulting from different runs of the admixture model.

**FIGURE A4:**
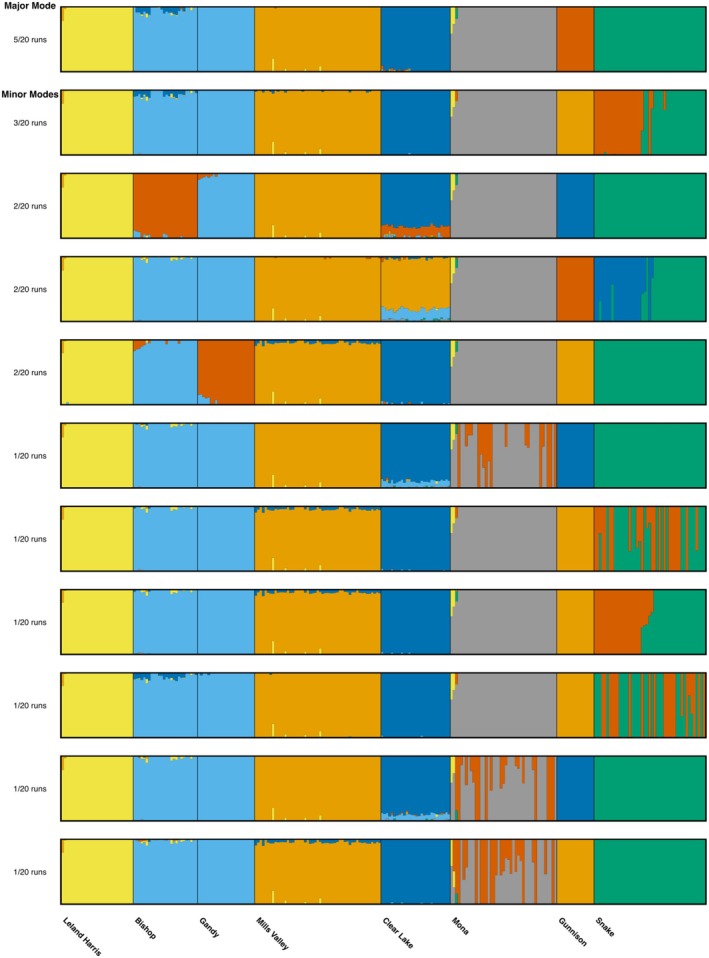
Admixture modes for *K* = 7 using output from the Stacks pipeline. Admixture proportion inference was run using ADMIXTURE v1.3.0 with 20 replicate runs. Variance in latent cluster membership was visualized using Pong v1.5. Modes indicate differences in cluster membership resulting from different runs of the admixture model.

**FIGURE A5:**
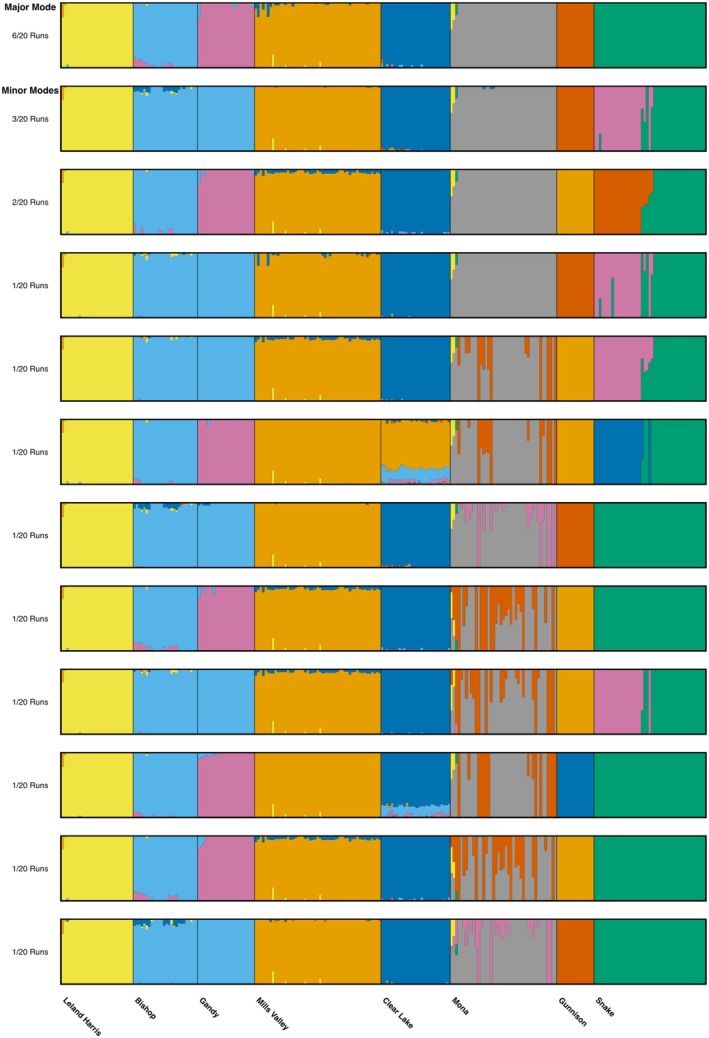
Admixture modes for *K* = 8 using output from the Stacks pipeline. Admixture proportion inference was run using ADMIXTURE v1.3.0 with 20 replicate runs. Variance in latent cluster membership was visualized using Pong v1.5. Modes indicate differences in cluster membership resulting from different runs of the admixture model.

Admixture analyses also showed distinct population structuring between population groups with little to no recent admixture (Figure 4). Cross‐validation (CV) indicated a *K* of 7 to contain the lowest error; though, CV error elbowed at *K* = 5 (Figure A1). Using the major modes of each *K* value, as *K* increased from 5 to 8, individual populations were moved to new groups, with Clear Lake separating from Mills Valley at *K* = 6, Gunnison separating from Mills Valley at *K* = 7, and Gandy separating from Bishop at *K* = 8. However, several different modes were present for each *K* value, with 5 modes at *K* values of 5 and 6, 11 modes at *K* = 7, and 12 modes at *K* = 8 (Figures A2, A3, A4, A5). Minor modes differed from major modes in the cluster placement of the Gunnison, Clear Lake, and Gandy populations, or by splitting the Snake River populations or the Mona population into two clusters. This uncertainty is likely due to the low levels of genetic diversity overall, high levels of diversity within populations, and the complex hydrologic history of the region. Of all populations, Clear Lake is the only population that exhibits potential recent admixture between Sevier River and West Desert GMUs; although, it is unclear if this is due to incomplete lineage sorting, recent bait bucket transfer, or a combination of the two.

**FIGURE 5:**
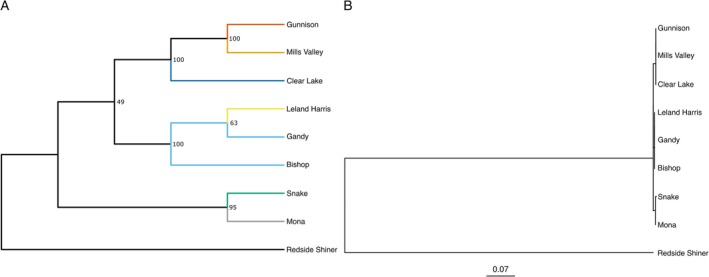
Consensus phylogenetic relationships of Least Chub from the Bonneville Basin in Utah and the upper Snake River in Idaho with Redside Shiner as the outgroup. (A) Cladogram displaying branching pattern of the tree. (B) Phylogenetic tree displaying estimated branch lengths. Trees were rooted on the outgroup and node support values were estimated from ultrafast bootstrapping with 1000 replicates. Populations are colored according to the Admixture analysis with *K* = 7 (see Figure 4).

Phylogenetic analysis showed distinct clades for the Sevier River (i.e., Gunnison, Mills Valley, and Clear Lake) and West Desert GMUs (i.e., Leland Harris, Bishop, and Gandy). Additionally, the analysis showed a clade containing Mona as sister to the Snake River populations (Figure 5); aligning with analyses for population structure. Overall, our results provide evidence that the Snake River populations and the Gunnison population represent natural, distinct populations of Least Chub, and reinforce that Clear Lake holds distinct genetic diversity.

## 4 Discussion

Through analysis of RADseq SNP data, we provide evidence that newly discovered populations of Least Chub in the Upper Snake River in Idaho and within the San Pitch River in Utah represent natural populations, greatly extending the known range of the species. Genetic diversity within our data reflects the general patterns of AFLP diversity observed by Mock and Miller (2005) and Mock and Bjerregaard (2007), with distinct population structure between the three GMUs in Utah. Additionally, we documented distinct population structure between the Utah populations and the Idaho populations. These results will be important for the management of the species by guiding the creation of new refuge populations and highlighting the need to search for additional unknown natural populations.

### 4.1 Genetic Differentiation Between Bonneville Basin and Upper Snake River Drainage

We have provided evidence for genetic divergence between populations in the Bonneville Basin and upper Snake River drainage indicating that Least Chub are a native species in the upper Snake River drainage. Least Chub in the upper Snake River were most closely allied to the Mona Springs population in the Bonneville Basin. This pattern is consistent with divergence patterns in other fishes shared between the basins, where more northerly populations in the Bonneville Basin are closely allied with populations in the upper Snake River (Johnson 2002; Mock et al. 2006; Billman et al. 2010; Houston et al. 2014). The divergence between these populations was sufficient to indicate that the Least Chub in the upper Snake River represent unique and native populations rather than originating from a bait‐bucket introduction. Consequently, the landscape for Least Chub conservation will change drastically as the geographic range of the species effectively doubles with this discovery.

The phylogeography of other fish species has demonstrated the importance of ancient connections of the Bear River between the Bonneville Basin and the upper Snake River. Phylogenetic studies for these species provided evidence that divergence between the upper Snake River and Bonneville Basin populations predates the Bonneville flood in the late Pleistocene (~15,000 years ago; Johnson 2002; Billman et al. 2010; Kokkonen et al. 2024). Rather, divergence patterns were consistent with more ancient connections with the upper Snake River and the Bear River prior to lava flows redirecting the Bear River into the Bonneville Basin ~100–50 kya (Bright 1967; Bouchard et al. 1998; Pederson et al. 2016). For example, recent studies have provided evidence that cutthroat trout in the Bear River are distinct from Bonneville Cutthroat Trout (*Oncorhynchus virginalis utah*) in the other portions of the Bonneville Basin and are more closely allied with Yellowstone Cutthroat Trout (*O. v. bouvierii*) in the upper Snake River (Campbell et al. 2018; Shiozawa et al. 2018; Kokkonen et al. 2024). Additionally, the Bonneville flood appears to have not influenced genetic divergence through admixture of Bonneville Cutthroat Trout that transferred into the upper Snake River drainage (Kokkonen et al. 2024). We were unable to identify similar patterns for Least Chub because we did not have any samples from the Bear River. However, we predict that Least Chub will demonstrate a similar divergence pattern that demonstrates the importance of ancient connections with the upper Snake and Bear rivers.

Connections between the Snake River and the Bonneville Basin were facilitated through the Bear River, suggesting that Least Chub were present in the Bear River historically. Currently, there are no known populations in the Bear River drainage. However, Least Chub were collected in the Bear River drainage near Logan, Utah, in 1918 (ANSP_50639; The Academy of Natural Sciences at Drexel University). Additionally, two collections of Least Chub from 1894 from the Bear River, Idaho, are in the Ichthyology Collection at the California Academy of Sciences (CAS‐SU[ICH] 24,272; CAS‐ICH 74569), but the coordinates for these collections place the samples from the Bear River in the Boise River drainage rather than the Bear River in the Bonneville Basin. Therefore, surveys that target Least Chub are necessary to determine if remnant populations are present in the Bear River drainage. Additionally, much of the potential geographic range of Least Chub in the upper Snake River drainage has yet to be surveyed for this species. Standard fish surveys conducted by management agencies in the Bear River and upper Snake River drainages primarily target salmonids and introduced sport fishes and are not likely to detect Least Chub (Billman et al. 2023). To evaluate the status of Least Chub in these regions, surveys that target the species are needed to understand distribution and abundance and to determine management actions that will be required to conserve remaining populations.

Interestingly, patterns of genetic diversity between Utah and Idaho populations differ between nuclear and mitochondrial markers. Mock and Miller (2005) demonstrated that mitochondrial DNA (Cytochrome b gene; cytb) showed low genetic variability in Least Chub. Billman et al. (2023) sequenced the cytb gene in 13 Least Chub from Henrys Fork Snake River (GenBank accession numbers: PV821485‐PV821497) and found three haplotypes: 11 individuals contained the common haplotype identified by Mock and Miller (2005) and seen in all populations of Least Chub, and two individuals each contained newly discovered haplotypes that differed from the common haplotype by one base pair. Whereas the SNP data indicate Idaho populations are most closely related to the Mona population, individuals from Henrys Fork Snake River do not share any of the unique cytb haplotypes seen in the Mona population (Mock and Miller 2005). As such, the historic timing and patterns of divergence in Least Chub are still unclear, though they were likely heavily influenced by changes in the hydrology of the Bear River and Pleistocene pluvial cycles (Williams et al. 2024). We suggest that future work beyond surveying the upper Snake River Drainage should focus on finding any potentially unknown populations in the Bear River Drainage (as mentioned above) and assembling a reference genome for Least Chub, which will allow more accurate estimations of historical demography using genotype‐free methods (Warmuth and Ellegren 2019).

### 4.2 Origin of Least Chub in San Pitch River

Populations of Least Chub in the Sevier River drainage demonstrated genetic structuring that provided evidence that the newly discovered Gunnison population in the San Pitch River is of natural origin rather than a bait‐bucket introduction. Least Chub in Mills Valley and Clear Lake represent unique populations that are closely allied to each other (this study; Mock and Bjerregaard 2007). Fish in the Gunnison population demonstrated similar divergence from Mills Valley as Clear Lake. Additionally, genetic structuring among these three populations followed their positioning along the longitudinal gradient in the Sevier River drainage. Genetic divergence was greatest between Least Chub in the Gunnison and Clear Lake populations, the upstream and downstream extent of known populations in the Sevier River drainage; the Mills Valley population was intermediate to the other two populations. Thus, the newly discovered Gunnison population should be managed as a unique, extant population.

### 4.3 Implications for Conservation of Least Chub

The landscape for conservation of Least Chub will need to change as we have provided evidence that the range of the species is double the long‐accepted range (Hanks and Belk 2004; Bailey et al. 2005). Additionally, the discovery of a previously unknown population in the San Pitch River provides evidence that more Least Chub may be present within the Bonneville Basin than currently documented. We recommend extensive surveys in both the Bonneville Basin and the upper Snake River drainage to further delineate the distribution of Least Chub and to identify new populations. These surveys will be essential for determining the status of Least Chub in the upper Snake River drainage to inform conservation strategies. Additionally, these surveys will inform habitat requirements for the species beyond the isolated springs to which studies have been restricted for over 50 years (Hanks and Belk 2004). Least Chub historically occupied streams and rivers in the Bonneville Basin (Bailey et al. 2005); however, the species was believed to be extirpated from fluvial habitats until their recent discoveries in the San Pitch River and in Henrys Fork Snake River (Billman et al. 2023). Conservation strategies for Least Chub have successfully focused on establishing refuge populations for the limited extant populations (Thompson et al. 2015). Knowledge gained from establishing refuges and from new surveys can also inform restoration of Least Chub into waterbodies where they have been extirpated (Bailey et al. 2005). Establishment of reintroduction protocols and prioritization of recipient waterbodies should build upon the experiences of similar programs for freshwater fishes (Williams et al. 1988; Dunham et al. 2011; Olden et al. 2011; Thompson et al. 2015).

Conservation of Least Chub will require additional studies to determine how different selective regimes across their distribution have contributed to local adaptation and ecological diversity. Continued studies on phylogeography, particularly if additional populations are discovered, can further elucidate the effects of drainage connections on genetic divergence of aquatic organisms in the region (Pederson et al. 2016; Kokkonen et al. 2024). Least Chub continue to persist in this region characterized by long‐term hydrological cycles that result in changing environmental conditions. The ecological and evolutionary processes associated with these changing conditions have given rise to ecological diversity within the fish species in the Bonneville Basin and the upper Snake River (Johnson and Belk 2007). Different selection regimes, isolation, and additive genetic variation influence local adaptation, which can affect variation in morphology, ecology, and life history (Johnson and Belk 1999, 2007; Houston and Belk 2006; Williams et al. 2017). Thus, future studies focused on Least Chub will help guide the conservation of this species and its unique ecological diversity. Additionally, this knowledge will determine how human activities interfere with ecological and evolutionary processes that have given rise to ecological diversity observed within Least Chub and other species in the region.

## Author Contributions

**Chance Broderius:** conceptualization (supporting), funding acquisition (equal), investigation (supporting), project administration (supporting), writing – review and editing (equal). **Paul Thompson:** conceptualization (supporting), funding acquisition (equal), investigation (supporting), writing – review and editing (equal). **Trevor J. Williams:** conceptualization (supporting), data curation (equal), formal analysis (lead), methodology (equal), visualization (lead), writing – original draft (supporting), writing – review and editing (equal). **Michael Terribilini:** data curation (equal), formal analysis (supporting), methodology (equal), visualization (supporting), writing – original draft (supporting), writing – review and editing (equal). **Cody Smith:** funding acquisition (equal), investigation (supporting), methodology (equal), writing – review and editing (equal). **Eric J. Billman:** conceptualization (lead), funding acquisition (equal), investigation (lead), project administration (lead), writing – original draft (lead).

## Conflicts of Interest

The authors declare no conflicts of interest.

## Data Availability

Raw sequencing data used in this project is available through NCBI's Sequence Read Archive (SRA) under accession number PRJNA1265784. Processed genotype data and the scripts used for analyses are available at Zenodo (https://doi.org/10.5281/zenodo.16229886) and GitHub (https://github.com/trevorjwilli/lc_radseq_2025). Reasonable requests for additional data can be fulfilled by contacting the authors.
